# The Earth BioGenome Project Phase II: illuminating the eukaryotic tree of life

**DOI:** 10.3389/fsci.2025.1514835

**Published:** 2025-09-04

**Authors:** Mark Blaxter, Harris A. Lewin, Federica DiPalma, Richard Challis, Manuela da Silva, Richard Durbin, Giulio Formenti, Nico Franz, Roderic Guigo, Peter W. Harrison, Michael Hiller, Katharina J. Hoff, Kerstin Howe, Erich D. Jarvis, Mara K. N. Lawniczak, Kerstin Lindblad-Toh, Debra J. H. Mathews, Fergal J. Martin, Camila J. Mazzoni, Ann M. McCartney, Nicola Mulder, Sadye Paez, Kim D. Pruitt, Verena Ras, Oliver A. Ryder, Lesley Shirley, Franç oise Thibaud-Nissen, Tandy Warnow, Robert M. Waterhouse, the EBP Community of Scientists, Alexandre Aleixo, Miguel Allende, Jonas Astrin, Miklós Bálint, Katherine Barker, Ian Barnes, Kathy Belov, Giorgio Bertorelle, Iliana Bista, Mark Blaxter, Tomas Marques Bonet, Irus Braverman, Titus Brown, Jing Cai, Nicolette Caperello, Juan Carlos Castilla Rubio, Shu-Miaw Chaw, Haidan Chen, Lei Chen, Anna K. Childers, Robert Cook-Deegan, Montserrat Corominas, Shannon Corrigan, Keith A. Crandall, Andrew J. Crawford, Manuela da Silva, Robert Davey, Alice Dennis, Federica Di Palma, Richard Durbin, Jay Evans, Samuel Eziuzor, Olivier Fedrigo, Marc Palmada Flores, Giulio Formenti, Nico M. Franz, Arthur Georges, Anita Ghansah, M Thomas P Gilbert, Melissa Goldstein, Henry T. Greely, Roderic Guigo, Kevin Hackett, Neil Hall, Peter Harrison, Uljana Hesse, Katharina J. Hoff, Carolyn Hogg, Kerstin Howe, Maui Hudson, Ozede Nicholas Igiehon, Sachiko Isobe, Kjetill Sigurd Jakobsen, Erich Jarvis, Rebecca N Johnson, Steven Jones, Nathaniel K. Jue, Elinor K. Karlsson, Sally Katee, Paul Kersey, Jin-Hyoung Kim, Kevin Kocot, Tiffany Kosch, W. John Kress, Josiah Kuja, Shigehiro Kuraku, Malathi Lakshmikumaran, Mara Lawniczak, James Leebens-Mack, Harris Lewin, Qiye Li, Xueyan Li, Kerstin Lindblad-Toh, Xin Liu, Jose V. Lopez, Jianguo Lu, Jian Ma, Meike Mai, Roksana Majewska, Ntanganedzeni Mapholi, Luisa S. Marins, Fergal J. Martin, Debra JH Mathews, Camila J. Mazzoni, Catherine McCarthy, Ann M McCartney, Duane D. McKenna, Phillip Morin, Anne WT Muigai, Gene Myers, Ellis C. O’Neill, Rachel J. O’Neill, Sadye Paez, Adam Phillippy, Monica Poelchau, Kim D. Pruitt, Verena Ras, Arang Rhie, Emillio Righi, Gene Robinson, Lily Rodriguez, Hugues Roest Crollius, Cristina Roquet, Oliver A. Ryder, Sunil Kumar Sahu, Cynthia Saloma, Bernardo Santos, H. Bradley Shaffer, Timothy M. Shank, Taukondjo Shikongo, Heitor Shimizu, He Shunping, Pamela Soltis, Cibele Sotero-Caio, Ciara Stauton, David Swarbreck, Boping Tang, Francoise Thibaud-Nissen, Bashir Bolaji Tiamiyu, Andrew Torrance, Krystal S. Tsosie, Marcela Uliano-Silva, Andrew Veale, Sonja Vernes, Olga Vinnere Pettersson, Kun Wang, Robert Waterhouse, Claudia C. Weber, Jill Wegrzyn, Xiaofeng Wei, Regina Wetzer, Jeremy Wideman, Jason Williams, Linda Wong, Charlotte J. Wright, Joseph M. Yracheta, Guojie Zhang, He Zhang

**Affiliations:** 1 Tree of Life, Wellcome Sanger Institute, Cambridge, United Kingdom; 2 Global Futures Laboratory, Walton Center for Planetary Health, Arizona State University, Tempe, AZ, United States; 3 Genome Center, Department of Evolution and Ecology, University of California, Davis, Davis, CA, United States; 4 Research and Innovation, Genome British Columbia, Vancouver, BC, Canada; 5 Department of Biological Sciences, University of East Anglia, Norwich, United Kingdom; 6 Fiocruz Biodiversity and Health Biobank, Oswaldo Cruz Foundation–Fiocruz, Rio de Janeiro, Brazil; 7 Department of Genetics, University of Cambridge, Cambridge, United Kingdom; 8 The Vertebrate Genome Laboratory, The Rockefeller University, New York, NY, United States; 9 School of Life Sciences, Arizona State University, Tempe, AZ, United States; 10 Computational Biology and Health Genomics, Centre for Genomic Regulation (CRG), Barcelona, Spain; 11 Department of Medicine and Life Sciences, Universitat Pompeu Fabra (UPF), Barcelona, Catalonia, Spain; 12 Barcelona Institute of Science and Technology (BIST), Barcelona, Spain; 13 European Bioinformatics Institute, European Molecular Biology Laboratory (EMBL), Hinxton, United Kingdom; 14 LOEWE Centre for Translational Biodiversity Genomics, Frankfurt, Germany; 15 Senckenberg Research Institute, Frankfurt, Germany; 16 Institute of Cell Biology and Neuroscience, Faculty of Biosciences, Goethe University, Frankfurt, Germany; 17 Institute of Mathematics and Computer Science, University of Greifswald, Greifswald, Germany; 18 Center for Functional Genomics of Microbes, University of Greifswald, Greifswald, Germany; 19 Neurogenetics of Language, The Rockefeller University, New York, NY, United States; 20 Howard Hughes Medical Institute, Chevy Chase, MD, United States; 21 Department of Medical Biochemistry and Microbiology, Uppsala University, Uppsala, Sweden; 22 SciLifeLab, Uppsala University, Uppsala, Sweden; 23 Broad Institute of MIT and Harvard, Cambridge, MA, United States; 24 Berman Institute of Bioethics, Johns Hopkins University, Baltimore, MD, United States; 25 Department of Genetic Medicine, Johns Hopkins University School of Medicine, Johns Hopkins University, Baltimore, MD, United States; 26 Berlin Center for Genomics in Biodiversity Research, Berlin, Germany; 27 Department of Evolutionary Genetics, Leibniz Institute for Zoo and Wildlife Research, Berlin, Germany; 28 Genomics Institute, University of California, Santa Cruz, Santa Cruz, CA, United States; 29 Computational Biology Division, Department of Integrative Biomedical Sciences, Institute of Infectious Disease and Molecular Medicine, Faculty of Health Sciences, University of Cape Town, Cape Town, South Africa; 30 National Center for Biotechnology Information (NCBI), National Library of Medicine, National Institutes of Health, Bethesda, MD, United States; 31 Department of Biodiversity and Conservation Biology, University of the Western Cape, Bellville, South Africa; 32 Conservation Science, San Diego Zoo Wildlife Alliance, Escondido, CA, United States; 33 Department of Ecology, Behavior and Evolution, School of Biological Sciences, University of California, San Diego, San Diego, CA, United States; 34 Department of Computer Science, University of Illinois, Urbana-Champaign, Urbana, IL, United States; 35 Environmental Bioinformatics, SIB Swiss Institute of Bioinformatics, Lausanne, Switzerland; 36 Department of Ecology and Evolution, University of Lausanne, Lausanne, Switzerland

**Keywords:** biodiversity, conservation, evolution, genomics, DNA sequencing, annotation

## Abstract

The Earth BioGenome Project (EBP) aims to “sequence life for the future of life” by generating high-quality reference genome sequences for all recognized eukaryotic species, thereby building a rich knowledge base to inform conservation, inspire bioindustry, ensure food security, advance medicine, and establish a deeper understanding of biodiversity. As the EBP works toward completing the original Phase I goal—a reference genome for each of the approximately 10,000 taxonomic families of eukaryotes—milestone publications have demonstrated the transformative potential of the project. The EBP has promoted global collaboration and established core methods and standards. By the end of 2024, EBP-affiliated projects had publicly released 2,000 high-quality genome assemblies, representing more than 500 eukaryotic families. In this article, we present a revised set of goals for Phases I and II of the EBP. For Phase II, we propose generating reference genomes for 150,000 species over 4 years, including representative genomes for at least 50% of all accepted genera and for additional species of biological and economic importance. To deliver Phase II, EBP-affiliated projects will have to release over 3,000 new genomes per month. We review the magnitude of the tasks in sourcing, sequencing, assembling, annotating, and analyzing genomes at this scale, and explore the scientific, technical, social, legal, ethical, and funding challenges associated with them. Success in Phase II will set the stage for sequencing the remaining ~1.5 million named species of Eukaryota and establishing the knowledge platforms necessary for understanding, preserving, and utilizing Earth’s biodiversity in an era of rapid environmental change.

**Key points:**

- The ongoing success of Phase I of the Earth Biogenome Project (EBP) demonstrates the feasibility of producing reference-quality genomes at scale, enabling the project to achieve its overarching goal: to sequence 1.67 million eukaryotic species in 10 years.
- Using knowledge from Phase I projects, we propose a revised strategy for Phase II: collecting specimens for 300,000 species and sequencing 150,000 species, representing at least half of the eukaryotic genera, in 4 years.
- Technical advances in DNA sequencing, genome assembly, and genome annotation have reduced costs and increased throughput to the point that we envisage globally distributed production of reference-quality genomes for most eukaryotic species for a total cost of about US$3.9 billion—US$800 million less than initially envisioned.
- Key challenges remain, including enhancing global coordination and building communities of users and interested parties; creating an inclusive, global biodiversity genomics workforce; developing effective access and benefit-sharing methodologies; facilitating collection at scale of vouchered specimens; sequencing reference genomes from single-celled and very small organisms; enhancing functional annotation; and building large-scale toolkits for comparative genomics.
- Technological and operational innovations, such as a “sequencing lab in a box,” have the potential to radically transform the global capacity for biodiversity genome sequencing, facilitating national benefit-sharing agreements and the realization of societal impacts on Indigenous peoples and local communities.
- We propose the establishment of a US$0.5 billion Foundational Impact Project (FIF) fund to support the immediate use of the genome sequences in conservation, agriculture, biodiversity monitoring, biotechnology, and basic sciences, focused on supporting initiatives in the Global South.

## The Earth BioGenome Project: past, present, and a new future

The Earth BioGenome Project (EBP; see https://www.earthbiogenome.org/) proposed a visionary goal: to sequence all named eukaryotic species in 10 years (1). This science “moonshot” is critical for future planetary and human health as it will transform our biological understanding of all life (2). The EBP has evolved as a network of networks that collectively engages local and global scientific, stakeholder, and public communities to generate a shared genomic resource to advance biodiversity science, underpin essential conservation efforts, and build a more equitable global bioeconomy (1, 3). The EBP originally planned to deliver this revolutionary change in three growing phases over 10 years. A completed 4-year pilot phase has built core methodologies, created standards, and established an ethical framework. In Phase I, which began in 2021, we proposed generating a high-quality reference genome sequence for most of the approximately 10,000 living eukaryotic families (3, 4). Here, we summarize progress in delivering Phase I goals and present a new vision for Phase II.

The EBP has formalized the organizational core of the project, helped to establish and recruit affiliated projects, created open governance principles, empowered committees to advise the project on technical and ethical standards, and planned workflows needed to produce reference-quality genome sequences at scale (5–7). Parallel, rapid advances in single-molecule, long-read, and high-throughput chromatin conformation capture (Hi-C) sequence data generation, as well as in the informatics of genome assembly, have made the production of high-quality, chromosome-scale assemblies much more achievable. In Phase I so far, EBP affiliates have delivered genomes at an inclusive average cost of US$28,000 per species assembled and demonstrated that high-quality genome assemblies can be generated from a wide diversity of taxa. These new genome sequences have been used to shine new light on fundamental and applied biological questions (8–15). Several large project consortia with strong buy-in from biodiversity, genomics, and end-user groups have been funded and started production, including the Vertebrate Genomes Project (VGP) (16), Bat1K (17), the Darwin Tree of Life Project (18), the African BioGenome Project (19), the Aquatic Symbiosis Genomics Project (20), the Norwegian Earth BioGenome Project (https://www.ebpnor.org/), the Catalan Initiative for the Earth BioGenome Project (21), the Canada BioGenome Project (http://earthbiogenome.ca/), the California Conservation Genomics Project (22), and the European Reference Genome Atlas (23) (see https://earthbiogenome.org/affiliated-project-networks). A live summary of EBP progress is maintained on Genomes on a Tree (GoaT; https://goat.genomehubs.org/projects/EBP) (24), an Elastic-search-driven data system organized against a taxonomic tree of all life from the United States National Center for Biotechnology Information (NCBI) Taxonomy database (25).

**Figure 1:**
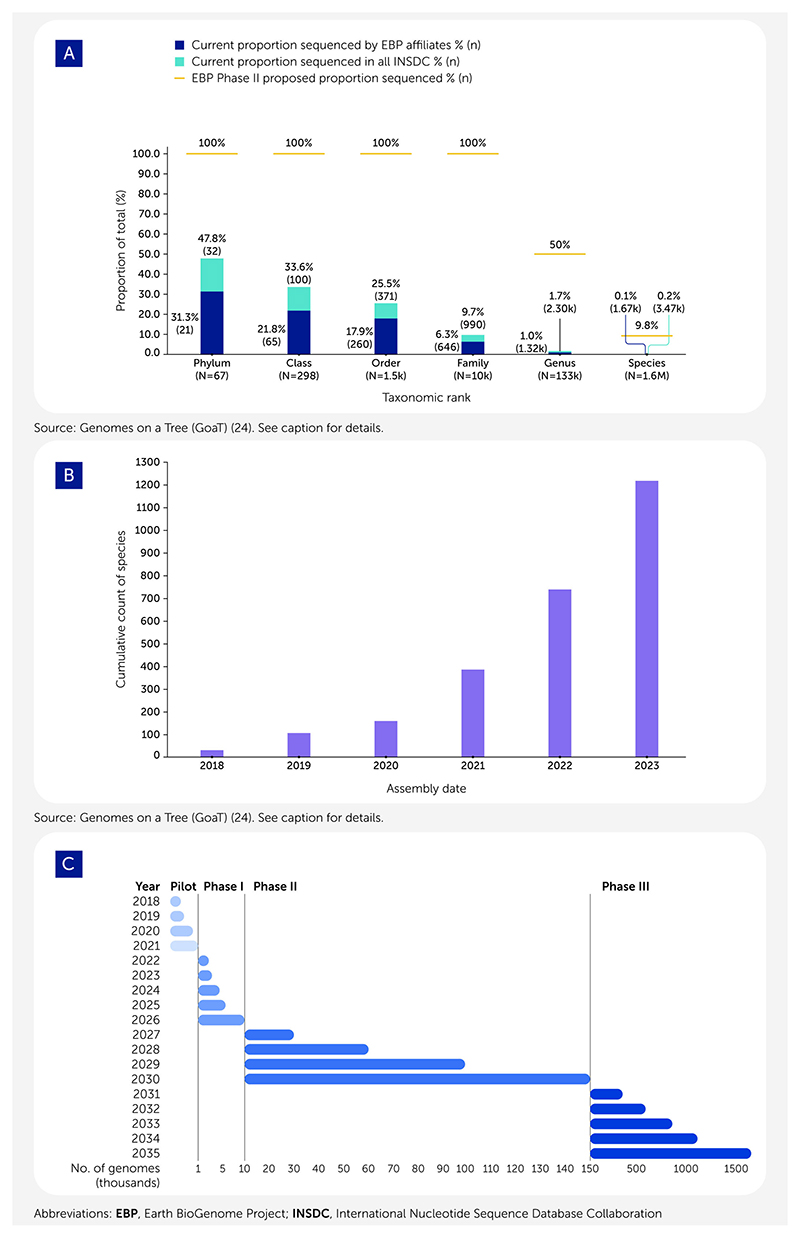
Progress toward sequencing all life in the Earth BioGenome Project (EBP). (**A**) The EBP’s goal of generating high-quality genomes across eukaryotic life is being realized. By September 2024, over 3,400 genomes with qualities meeting the EBP minimum contiguity standards (contig N50 >1 Mb, scaffold N50 >10 Mb, and >95% of the genome in chromosomal super-scaffolds) had been made available in the open International Nucleotide Sequence Database Collaboration (INSDC) databases, representing nearly 48% of all phyla and nearly 10% of all families (turquoise histogram bars). Of these high-quality genomes, 48% have been generated by EBP-affiliated projects (blue histogram bars). EBP Phase II goals (gold lines) are shown. At the end of Phase II, the EBP aims to complete the sequencing of nearly 10% of all species and the vast majority of all families. Plot based on data presented in Genomes on a Tree (GoaT) using the United States National Center for Biotechnology Information (NCBI) Taxonomy database’s taxonomy (see https://goat.genomehubs.org/2024.09.14/) (24). (**B**) The histogram illustrates the accumulation of EBP-standard genomes available in INSDC databases sorted by year of release. Plot based on data presented in GoaT (see https://goat.genomehubs.org/2024.09.14/) (24); assembly-level classification follows the INSDC definitions as outlined at https://www.ncbi.nlm.nih.gov/assembly/help/. (**C**) A timeline for EBP Phases I, II, and III is shown, indicating the approximate timing of each phase in terms of genome sequence delivery. The goals for Phase III are provisional and dependent on the success of Phase II.

The original plan for EBP Phase I was to deliver approximately 10,000 genome sequences, one for each eukaryotic family, over a 3-year period (an average of 300 genomes/month). As of September 14, 2024, EBP-affiliated projects had generated 1,667 high-quality genome sequences from fungi, plants, animals, and diverse protists, that met the minimum EBP reference genome metrics (generally 1 Mb contig N50, chromosome-scale scaffolds for all chromosomes with >95% of all sequence in chromosomes, and a base call error rate of less than 1/10,000, summarized as “6.C.Q40”; see https://www.earthbiogenome.org/report-on-assembly-standards). Other researchers deposited 1,798 EBP-quality genomes in International Nucleotide Sequence Database Collaboration (INSDC) databases (Figure 1A). Global production by EBP-affiliated projects was approximately 50 genomes/month in 2023 (see https://tinyurl.com/EBP-by-month-2023-in-GoaT) (Figure 1B). This was double the output in 2022 but is still insufficient to complete the Phase I goal in 3 years.

Our experiences in Phase I have revealed both strengths and limitations in our original strategy. There are still challenges to overcome to complete the goals of Phase I, which will be amplified in Phase II. The more ambitious EBP-affiliated projects have shown that genome sequencing across diversity can be achieved at scale by optimizing all aspects of the sequencing process, from sampling to assembly curation (6, 16, 26, 27). Individual advances are small, but they sum to a significant step-change in genome production. What is clear is that, given funding, EBP-quality reference genomes can be produced at scale, regardless of whether the target is a protist, a fungus, an animal, or a plant (see the Darwin Tree of Life Genome Notes collection for examples of successful chromosomal assembly of specimens of all these taxa; https://wellcomeopenresearch.org/gateways/treeoflife). The main factor limiting reference genome production has been funding, although more than US$200 million has already been raised. Knowledge gained to date, coupled with rapid advances and cost reductions in DNA sequencing, have led us to revise the staging and quality goals of future EBP strategy (Figure 1C). In a revised strategy for Phase I, we will sample from all phyla and from at least 50% of families. In Phase II we propose sequencing, to reference quality, 150,000 additional species, down from 180,000 species. We recognize that collecting strictly to a species list is inefficient, and specimens for many genera, such as deep-sea taxa, may be too cost-prohibitive to acquire. Instead of sequencing one species per genus, as originally proposed, we will sequence representative genomes for at least 50% of genera (80,000 species). We will also prioritize sequencing species of importance to ecosystem health, food security, pandemic control, conservation, and Indigenous peoples and local communities. Importantly, we propose that sequencing to reference quality, rather than draft, should be our goal. In completing Phase II, we will have sequenced about one-tenth of the Earth’s known eukaryotic biodiversity.

While many challenges and blockers to completion of Phase I overlap with those of Phase II, the scaling required to sequence 150,000 genomes in 4 years presents unique scientific and social challenges. While Phase II remains challenging, we are optimistic that our goals are achievable and that the data will be transformative.

### A new EBP Phase II strategy

The EBP is a progressive project, with overlapping rather than stepwise phases. A five-fold increase in reference genome output rates is needed to achieve the goals of Phase I, and a further 10-fold increase is required to deliver Phase II (over 3,000 genomes per month) (Figure 1C). To deliver this increase, the Phase II proposal is built around three pillars: adaptive sampling, highest genome quality, and equitable global partnerships.

### Pillar 1: Adaptive sampling

Phase II should collect and biobank samples from 300,000 species and sequence 150,000 of these. Sampling will follow the evolving and exacting technical, ethical, and legal standards established during EBP Phase I (https://www.earthbiogenome.org/sample-collection-processing-standards-2024). An adaptive species selection strategy will maximize the number of genera sequenced while also delivering genomes for species that are economically and ecologically important, iconic, of special scientific interest, or of cultural significance to Indigenous peoples and local communities (with their assent).

### Pillar 2: Highest genome quality

Given the radical changes already achieved in genomic technologies, and the promise of further improvement to come, we propose that as many as possible of the 150,000 Phase II genomes be sequenced to EBP reference quality (https://www.earthbiogenome.org/report-on-assembly-standards). Generating genome sequences of high quality will transform their impact both as references for a focal species and collectively across ecosystems, major groups, and the entire field of biology. The technical challenges of generating reference genomes to current EBP standards for microbiota and meiobiota must be met and overcome.

### Pillar 3: Global leadership through equitable partnerships

It is imperative that the EBP has a global base, equitably distributing sample acquisition and data-generation activities and ensuring the equitable realization of the benefits of the work and the resources. To achieve Phase II, much of the species’ collection, sample management, sequencing, assembly, annotation, and analysis will have to be based in the Global South and be delivered by EBP partners based in those nations. Genome sequencing will need to be supported at multiple sites in the Global South, including, especially, at laboratories based and sustained in countries with high biodiversity. We propose establishing a Foundational Impact Fund (FIF) to catalyze the realization of these benefits.

These three pillars will also be fundamental to Phase III. By building an equitable global network of cooperating partners promoting best practices in engagement and benefit-sharing, establishing rigorous standards and reproducible methods for the acquisition and sequencing of specimens, and inventing new methods and systems for large-scale annotation and analysis of many thousands of genomes during Phase II, we will be well placed to generate the genomes of all named eukaryotic species on Earth in Phase III.

### What should we sequence?

The EBP ultimately aims to generate reference genome sequences for all 1.67 million named eukaryotic species at the time of this writing—the species formally described by taxonomic communities over the last centuries (1, 4). The precise number of species known on our planet increases as new species are discovered and decreases as species become extinct and taxonomic revision resolves synonymy. Indeed, species are being described at accelerating rates, in part driven by an emerging synergy between molecular, morphological, and machine-learning (ML) approaches to taxonomy (28, 29). The total number of extant species is much greater than those described, with a consensus that we share our planet with at least 10 million other eukaryotes (28, 30–33). While genomics will play a significant role in the discovery and description of new species (34, 35), the EBP will continue to focus on named eukaryotic taxa rather than attempting to sequence and diagnose the vast number of undescribed species.

Not all species are equally accessible for collection and sequencing. We will adaptively prioritize species for sequencing in Phase II using the following four principles in sampling.

- **Phylogenetic diversity:** Phase II will select species representative of previously un- or under-sampled parts of the eukaryotic tree of life. Practically, this can be achieved by aiming to sample a representative for all accessible families (Phase I) and all accessible genera (Phase II).
- **Conservation:** Phase II will ensure that species subject to conservation efforts, such as the >47,000 species on the International Union for Conservation of Nature (IUCN) Red List of Threatened species, are among the first to be sequenced. A process that allows communities, including representatives of Indigenous peoples and local communities, to nominate species for sequencing will promote engagement and understanding of genome sequencing and the potential value of its outcomes.
- **Ecological or societal impact:** individual species can play keystone roles in the ecosystems in which they thrive. Species can also be important to human society because they provide ecosystem services, food, or other biomaterials or are pathogens, pests, or predators of valued species. Phase II will contribute to planetary health goals by prioritizing the sequencing of these species.
- **Exceptional biological interest:** genomic sequencing can be a foundational step in understanding biology. For example, by sequencing species that appear to defy fundamental rules of biology, we can gain a deeper understanding of these rules. By sequencing all species in a well-studied ecosystem, we will better understand the interactions and dependencies that shape and maintain biodiversity.

**Figure 2:**
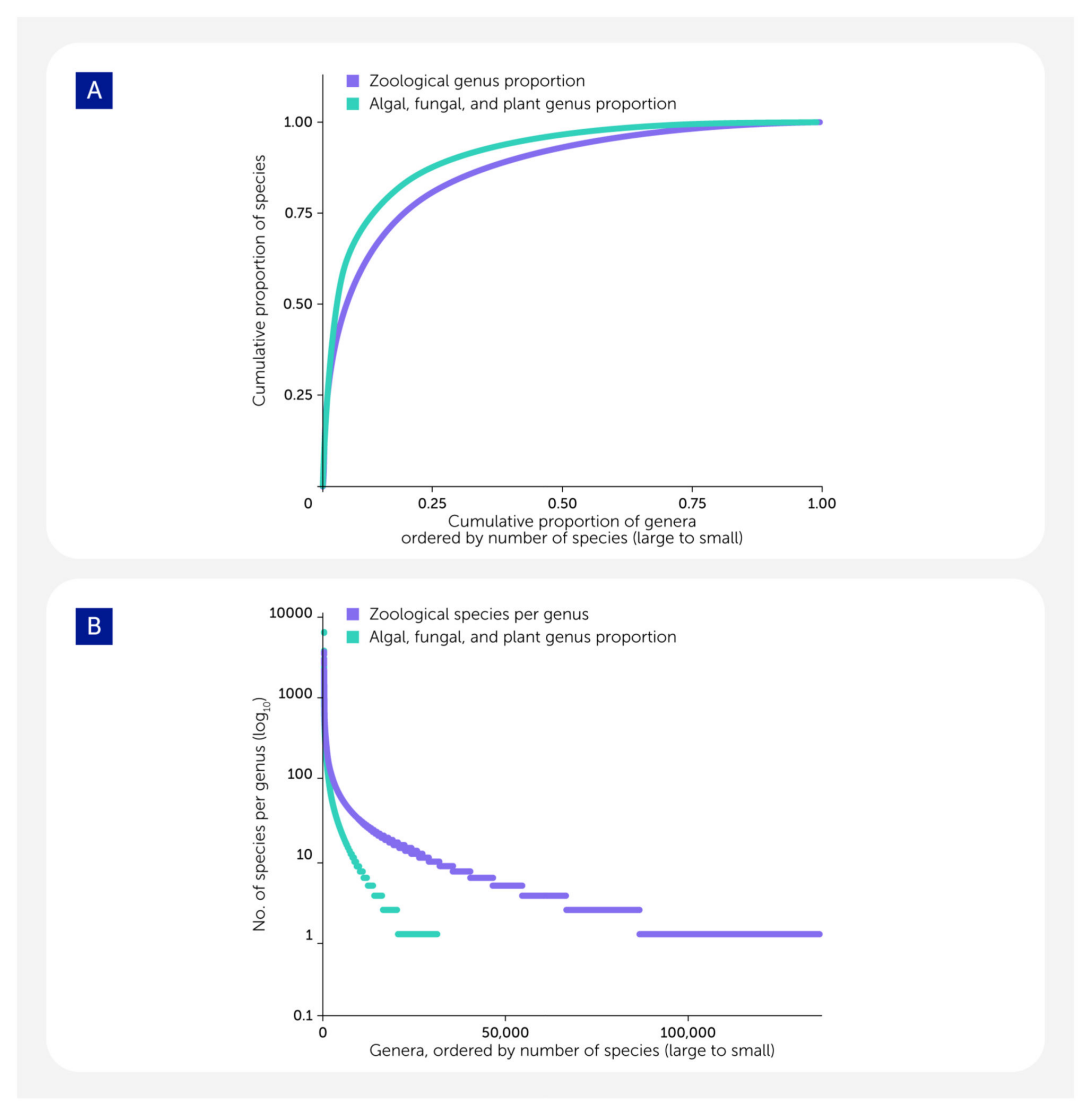
The pattern of life’s diversity. (**A**) Less than 5% of all genera contain 50% of all eukaryotic species. For species defined under the International Code of Zoological Nomenclature (ICZN; see https://www.iczn.org/the-code/the-code-online/), 4.2% of genera (5,742) contain 50% of described animal species, and for species defined under the International Code for algae, fungi, and plants (ICNafp; see https://www.iapt-taxon.org/nomen/main.php); 2.6% of genera (807) contain 50% of described plant, fungal, algal, and other protist species. (**B**) Most genera contain only one or two species. Plot of the number of species per genus; genera are ordered by the number of species they contain. The most speciose genus defined under the ICNafp is *Hieracium* L. (hawkweeds, 5,524 species), while under the ICZN, the most speciose genus is *Stenus* Latreille (semiaquatic rove beetles, 3,113 species). The analyses presented are based on data available from the Catalog of Life on 31 December 2023 (4). The processed data are available in the Supplementary material.

Simply finding specimens is a key challenge. Species range from widespread to localized, common to rare, and large-bodied to small. The easiest to sample and sequence are those that are widespread, common, and large. The local, rare, and small are challenging to find, identify, *and* sequence. Based on centuries of biodiversity research, accelerated by the recent digitization of species’ occurrence records, we have a reasonable overview of global eukaryotic diversity and its distribution, collated, for example, in the Global Biodiversity Information Facility (GBIF; see https://www.gbif.org/). Collecting representatives of every one of the approximately 167,000 living, valid eukaryotic genera (4) in just 4 years is infeasible. About one-third of all genera have only one or two species (Figure 2), and many of these are rare. Many species have been observed once and never recollected, and, sadly, many may be the victims of hidden extinction (36). While we fully expect that many collections made for the EBP will include chance encounters with rare species, it is clear that many other rare species will be practically uncollectable.

For Phase II we propose collecting 300,000 species, twice as many as will be sequenced. The species unsequenced in Phase II will prime Phase III. Campaigns focused on particular taxa (e.g., the VGP or Bat1K) (16), on species of particular concern (e.g., the Australian Threatened Species initiative; see https://threatenedspeciesinitiative.com) (37), on particular modes of life (e.g., the Aquatic Symbiosis Genomes project; see https://www.aquaticsymbiosisgenomics.org/) (20), or based in “genome observatory” sites delivering ecologically linked suites of species for sequencing (22, 38) will be critical in driving synergy between large-scale genomic sequencing and societal, ecological, and community benefits.

### Challenges, blockers, and proposed solutions to achieve EBP Phase II

We have identified many technical and social challenges that must be overcome to realize the Phase II goal of collecting 300,000 species and producing 150,000 high-quality, annotated genome assemblies from projects worldwide. Below, we present the technical challenges as five interrelated themes:

(1) coordinating the sampling of 300,000 species in 4 years,
(2) progressing from sample to sequence to assembly at an increasing scale,
(3) producing high-quality annotations of 150,000 genomes,
(4) delivering impactful analyses,
(5) integrating innovative, planet-friendly informatics.

We also discuss the enormous challenges in creating a global biodiversity genomics workforce, coordinating such a large project across the planet, and securing funding. Many challenges have a cross-cutting impact and solutions require close collaboration between experts in many domains.

### (1) Coordinated sampling of 300,000 species in 4 years

Sampling 300,000 species presents a set of interlinked organizational, logistic, technical, educational, and social challenges. We must build an adaptive species sampling strategy informed by taxonomic, geographic, and prioritization considerations—and which is legal, ethical, politically sensitive, and culturally aware—and align it with the overlapping constraints and drivers of partner engagement and the availability of local or international funding. Data systems that aggregate biodiversity data, such as GBIF and GoaT, will facilitate the sharing and coordination of EBP Phase II activity.

#### Building a global community rooted in local action

The EBP was envisaged as a hubs-and-spokes organization of regional nodes and taxon-focused projects (1). The human division of the planet into nation-states does not overlap with the ecosystems, biomes, and bioregions that pattern biodiversity. Stewardship of biodiversity is similarly localized, and individuals and groups, including Indigenous peoples and local communities, have a deep local understanding of species diversity (5). The EBP will have the greatest impact if we build on these strong, local foundations. Here, we present a model for EBP regional nodes, based on building autonomous capacity for genomics, from sample acquisition to genome analysis. We emphasize that, in addition to *collecting* locally, we envision regional nodes that will also *sequence, assemble*, and *analyze* locally.

The throughput required to meet EBP Phase II goals could be delivered by 25 regional nodes, each collecting an average of 12,000 species and sequencing and assembling at least 6,000 species over a 4-year period. The inception of regional nodes will be driven by local initiative, availability of funding, and assessment of accessible biodiversity. Regional nodes will build on existing local scientific collaborations and knowledge. Sustainable regional nodes will require local skills, capacity, and funding (see the Workforce section below) and will likely take 2 to 3 years to implement.

Sample acquisition relies heavily on human capital and local skills. In contrast to expected savings in sequencing and assembly, as harder-to-source species are targeted, the costs of collecting will be relatively static per species. We envisage only a 40% reduction between Phase I and Phase II, even with the implementation of novel technologies. Much of the required expertise resides in local learned societies, taxon interest groups, national and local biological collections, and Indigenous peoples and local communities. We propose formal recruitment of collector allies to each regional node, who will bring specific taxonomic or habitat expertise and local user-community agendas. To promote sustainable careers, allies could agree to provide specified sets of legally and ethically sourced species, receive guaranteed compensation to recover staff and other costs, and be awarded explicit scientific credit for their work. Species acquisition for EBP sequencing through allies will support the currently underfunded expert taxonomy community, build capacity, and promote engagement with conservationists and other practitioners with the goals and outputs of the EBP.

It is essential that regional nodes should be established in biodiverse regions, especially in the low- and middle-income nations of the Global South, which have historically been underrepresented in or excluded from the global scientific commons. New regional nodes should be strongly supported by existing biodiversity genomics centers. Building on existing installed capacity and interest, this will establish a legacy of genomics expertise that can be leveraged for a range of post-genomic work, including FIF projects. One way such capacity could be achieved is through the installation of a complete “genomes from a box” (gBox), specimen-to-sequence laboratory (see Box 1), equipped for EBP data production at scale. A gBox install would be accompanied by support from other established nodes through a system of mutual aid and buy-in from technology companies for reagents and support.

For Phase III, both of these models (regional nodes and biodiversity genomics allies) will have to be expanded to ensure collection from all biomes in an inclusive, just, and ethical manner. Regional nodes will serve as focal points to usher in a post-EBP world of genome-enabled science for conservation, medicine, and bioindustry.

#### Assuring delivery of the highest-quality samples to make the highest-quality genomes

EBP specimens contributing to reference genome assemblies should be accompanied by rich metadata. From sample acquisition to genome publication, the EBP will enforce use of the GBIF Darwin Core standards, which define information that must accompany any globally aggregated biodiversity data record (https://www.gbif.org/standards) (39). This will be coordinated via Darwin Core-compatible metadata management systems such as Symbiota (40). The EBP will redouble efforts to make specimen metadata, genomic data, and analyses compatible with the (sometimes conflicting) demands of the FAIR [Findable, Accessible, Interoperable, and Reusable (41)] and CARE (Collective benefit, Authority to control, Responsible, Ethical) (https://www.gida-global.org/care) (42) principles of data governance. Wherever possible, a Traditional Knowledge and Biocultural Label or Notice (https://localcontexts.org/) should be attached.

Methods for high-quality, three-dimensional (3D) imaging of specimens compatible with the use of specimens for genomics are sorely needed. These images would provide an essential digital voucher for specimens subsequently consumed for sequencing. Imaging will be critical as the project progresses, as confidence in species identity may be lower, and sequencing may occur before full taxonomic identification. EBP specimen images could contribute to training resources for artificial intelligence (AI) and other computer-aided species identifications (28, 43, 44). The development of open-data systems and smartphone applications, similar to or allied with the popular iNaturalist platform (https://www.inaturalist.org/), would provide significant benefits.

Biobanking is critical for storing materials for future analyses. Detailed recommendations and protocols for biobanking in the age of genomics are available (45). Regional nodes must put in place secure biobanking of samples, ideally in collaboration with national or regional museums, botanical gardens, and other collections. Expansion of biobanking to explicitly support additional modes of analysis, such as proteomics, metabolomics, and single-cell atlasing, is recommended. Whenever possible, and especially for endangered species, cell lines should be created for future conservation efforts (46, 47).

Currently, DNA and RNA extractions maximally compatible with high-quality genomics are achieved from fresh or ultra-low-temperature flash-frozen material. Best practices for sample acquisition and shipping currently rely on live transport or an unbroken cold chain from collection to extraction. These practices are unsustainable on a global scale based on logistical, welfare, cost, and environmental grounds. Approaches that preserve specimens at ambient temperatures will be game-changing in terms of expanding sample collection. These are being explored with some success (48) but remain an urgent development area.

Delivery of EBP goals will require contemporaneous processing of several thousands of species in a single laboratory. Robust laboratory information management systems and electronic lab notebooks are essential. Live aggregation of sample process data from these tools in a workspace such as GoaT (24) would enhance shared learning of best practices. EBP members are already using open platforms to share best practices for collection, storage, and extraction (e.g., through protocols.io; see https://www.protocols.io/workspaces/earth-biogenome-project). EBP Phase II partners can enhance the content of these platforms with protocols modified to work at scale across diverse species.

### (2) From sample to sequence to assembly at increasing scale

To achieve the EBP Phase II goal of 150,000 reference-quality genomes in 4 years, the affiliated projects will have to sequence, on average, 3,125 genomes per month. To deliver this throughput, the EBP must recruit many additional data generation sites in regional nodes and other centers. However, we must also develop improved sequencing and assembly processes, particularly methodologies that enable automated laboratory workflows and improved bioinformatics workflows. Algorithms that better exploit the richness of long-read and long-range data, for example, in building fully haplotype-resolved assemblies, will be essential. The sequencing of single-celled and meiobiotal eukaryotes and the separation of target species from potential cobionts will be more challenging, but successes in these areas are already promising (8, 49–51). These developments will also prime the EBP for Phase III.

#### Defining and meeting high-quality genome standards

Following the lead of the VGP (16), the EBP has established exacting but achievable quality metrics for reference assemblies (https://www.earthbiogenome.org/report-on-assembly-standards). We note that it is currently not technically possible to generate assemblies that meet these metrics for some species, largely because of small organism size and, consequently, minimal yield of DNA. Assemblies of such species will be attempted and submitted to the public databases, aiming to meet the EBP *representative* metric (0.1 Mb contig N50, chromosomal level, and 6.C.Q40). On the other end of the quality spectrum, we expect complete and near error-free, i.e., telomere-to-telomere, assemblies for a growing number of species, where contig N50 is the same as chromosomal N50, and all chromosomes are complete (C.C.Q40) (16, 52, 53).

The current recipes for genome sequencing to meet EBP metrics involve a mix of cutting-edge technologies. Three data types are currently used: single-molecule long-read data for contig building, long-range data from Hi-C for scaffolding, and transcriptomic data for accurate annotation. Close collaboration with technology providers will be essential to generate these data types at reduced per-genome costs (Table 1, see section, Costing the new Phase II strategy). These savings must be available worldwide and include equity-based price reductions.

In the future, it may be possible to simplify sequencing so that high-quality genome assemblies can be generated from a single data type, and workflows can be simplified by running long-read, long-range, and transcriptome libraries together on a single platform. Applying this paradigm (one sample, one library, one run, one genome) across biodiversity would put the EBP in a very strong position to deliver Phase III genomes to reference quality. New data types may also prove to be useful. It is already clear that ultralong reads (>100 kb) can be used to deliver much more contiguous, true telomere-to-telomere assemblies (54, 55). The generation of such data for a significant fraction of EBP target species would elevate the quality and value of the genomes produced.

#### Overcoming technical challenges to genomics for all biodiversity

Sequencing and assembly procedures for most taxa are robust and ready for Phase II implementation (16, 26, 56), but challenges remain. We estimate that for about half of extant species, less than 1 ng of DNA can be isolated from a single specimen, orders of magnitude less than the input requirements for many current long-read processes. We need to develop robust, transferable protocols that generate genomic sequencing libraries from minuscule inputs without compromising assembly quality. Some success in this area has already been reported (8, 49, 50). More challenging still is the sequencing of single-celled eukaryotes, including the paraphyletic “protists” and some fungi, several of which have surprisingly large genomes. Some will be sequenced from clonal cultures, but most species are not in culture. EBP-standard genome sequencing of single cells from environmental sources is currently very challenging. Approaches that combine bulk and single-cell data are promising (57–60) but need further development. EBP-affiliated projects are also exploring the problems presented by polyploid genomes, where varying levels of rediploidization make the assembly of distinct sets of homeologous chromosomes difficult (52, 61).

Another issue that impacts biodiversity genomics based on sampling from the wild is that target organisms may be accompanied by mutualist or parasitic symbionts, components of the host microbiome, or by accidentally co-isolated organisms (51, 62). Robust separation of the genomes of these cobionts from that of the target species is essential to avoid misattribution of biological capacity. This work has been facilitated by the recent development of highly sensitive and specific decontamination workflows such as Foreign Contamination Screen (FCS)-GX (63). Nevertheless, close attention to this aspect is essential for the future.

Informatics workflows covering primary assembly, haplotypic duplication removal, scaffolding, decontamination, and pre-curation processing, readying for EBP Phase II, are already openly available in workflow management systems such as Galaxy (64) and Nextflow nf-core (e.g., https://pipelines.tol.sanger.ac.uk/pipelines). The use of AI and ML toolkits to intelligently automate decision-making processes, such as raw data quality control and assembly curation, will make the flow of genomes more efficient and improve output genome quality. Equally important will be the open sharing of process and quality control information, so that issues can be foregrounded and solutions found rapidly for all EBP nodes.

Delivery of these advances will require extensive, focused research and development in the academic and commercial sectors to develop better methods of acquisition and shipping of specimens, extraction of nucleic acids, sequencing, and assembly. We have included an estimate of US$100M for these activities in each of Phases II and III.

### (3) High-quality annotation of 150,000 genomes

Annotation is crucial for understanding the functions encoded in a genome and is the starting point for downstream analyses (65). Over the last decade, major annotation services such as Ensembl (66) and RefSeq (67, 68), and the broader annotation community (69–72) have increased the quality and speed of annotation. Despite these successes, annotation remains a complex and computationally expensive process and a bottleneck to unlocking the value stored in genomes. To meet Phase II goals of annotating 150,000 genomes over 4 years, we need to develop radical new annotation approaches that leverage the diversity of expertise across the global community and optimize the use of available data and computational resources. In particular, new, scalable approaches should be supported, such as cross-genome orthology predictions—as used in TOGA (Tool to infer Orthologs from Genome Alignments) (72)—and AI deployment in gene prediction (73). As currently unexplored branches of the tree of life are illuminated by new reference genome assemblies, we expect to find exceptions to general patterns derived from the current relatively small subsample of annotated genomes (>5,000 eukaryotes, mostly yeast), such as new genetic codes, diversity in splicing mechanisms and patterns, and programmed editing during transcription. These “exceptions that prove the rule” can be identified, defined, and deployed to better understand the functional genomics of all life.

#### Annotating genomes to realize their value

The minimal annotation product for every EBP species should be the annotation of protein-coding and conserved non-coding gene types (https://www.earthbiogenome.org/report-on-annotation-standards) (6), accompanied by repeat finding using curated repeat libraries and *de novo* discovery. Many transcription units, perhaps most in lineages such as vertebrates, give rise to more than one mature transcript, and defining the diversity of these isoforms is essential to unpicking the true diversity of genes. Currently, all annotation approaches rely fundamentally on alignment to the genome of transcriptome or protein data, and statistical models of genomic features. For EBP Phase II, the generation of at least 50 million read pairs of short-read transcriptomic data from a single library for every species is the absolute minimum for high-quality annotation. Long-read transcriptomic data are an attractive alternative to standard short-read RNA sequencing as they robustly reveal the diversity of transcript isoforms (74), but are currently expensive compared to short-read data. Concatenation sequencing of full-length complementary DNAs (cDNAs) on the Pacific BioSciences (PacBio) high-fidelity (HiFi) long-read sequencing platform promises to deliver sequence reads for annotation at a reasonable cost (75), and transcript normalization may maximize the utility of these data for annotation. Development and community benchmarking of these and other techniques may usher in an expectation of long-read transcriptomics as standard. High-quality annotation, such as that currently built for “model” species, relies on transcriptome data covering multiple tissues, developmental stages and conditions, and additional functional genomic data. This is unlikely to be achievable for most species, where developmental stages are not collected and dissection into tissue types is impractical. It will be important to build tools that recognize the diversity of alternative splicing, perhaps using information from related species.

#### Beyond just transcriptomics

Cells and organisms read their DNA code using a complex mix of sequence-based and epigenetic signals. The development of AI toolkits to predict genes and their likely activity will need significant functional data beyond just deep sampling of mature messenger RNA (mRNA) transcripts. The EBP encourages the generation of additional modalities of functional genomic data for which high-throughput methodologies are already available (such as sequencing non-polyadenylated RNAs and small RNAs, identifying transcription start sites, determining cytosine and adenine DNA methylation, mapping open chromatin, and defining the patterns of chromatin histone modification), especially for species representative of families. High-quality annotations driven by rich multimodal datasets for a diverse set of carefully chosen species across biodiversity would provide a platform for new comparative approaches that leverage whole-genome alignments between well-annotated and newly sequenced species to simultaneously annotate coding genes and infer orthologous gene loci (72). AI offers an emergent opportunity to achieve high-quality annotation, particularly of protein-coding loci, by “learning” the embedded transcriptional code from well-studied taxa (73). Sharing existing and new high-quality, curated, and transcriptome-validated gene predictions as dense training data for deep learning approaches will be imperative to promote these developments.

Annotation of EBP genomes should aim to meet FAIR and CARE principles and be made publicly available through submission to INSDC databases. FAIR principles demand that annotations should be accompanied by defined metadata, including methods (software tools and parameters), external data used, and agreed-upon quality metrics. Tools such as Benchmarking Using Single Copy Orthologs (BUSCO) (76), compleasm (77), and OMArk (78, 79) that exploit the expectation of the presence of a curated set of single-copy orthologs to assess coding-gene annotation completeness will need to be dynamically updated to maintain precision as Phase II ramps up. Additional quality assessment metrics require development, such as descriptors of ancestral linkage group retention, gene structure congruence, the proportion of genes with transcriptome support, and the number of proteins containing known domains. A standardized tool for multidimensional metric computation from a genome and associated annotation files would contribute to streamlining the entire process of producing reference-quality genomes across the eukaryotic tree of life.

### (4) Delivering impactful analyses

To realize the value in EBP genome assemblies, we need to re-envision how we derive meaning from genomic data at scale. The EBP will, collectively, generate exabytes of raw and analyzed data, and Phase II alone will generate in excess of 200 terabases of assembled genomes. While this wealth of data promises a time of plenty for analytical genomics, it also brings challenges. The drive in the EBP for uniform, high-quality, and open reporting and assessment of assembly metrics will promote the combinability of all the genomes released. In comparative genomics, many computational tasks scale unfavorably with the number of genomes analyzed. Addressing these bottlenecks will require the development, coordination, and integration of research tools, infrastructure, and human resources at an unprecedented scale. The proposed FIF is designed to facilitate these analyses, especially by supporting initiatives, researchers, and organizations in the Global South.

#### Comparative and conservation genomics at an unprecedented scale

For comparative genomics analyses, the EBP will have to ensure that products derived from genome sequencing are available for open use. Products will include large-scale whole genome alignments, ancestral linkage group inference (10, 80, 81), repeat and mobile element family data aggregated across species (82), up-to-date and comprehensive gene orthology calls (78), genome-anchored descriptions of conserved functional elements (83–85), and genome-wide description of the 3D structure of each genome (86). To generate these at the new scale of Phase II will require active development of the toolkits used, many of which can currently only scale to tens or hundreds of genomes. The EBP must foster and, where possible, sponsor the exploration of new algorithms and computer architecture for comparative analyses, promoting the inclusion of all data-generating communities and nations.

Globally accessible resources are particularly important for species and ecosystems under threat, where targeted investment in additional data, such as population genomics data, will be crucial in estimating extinction risk, managing wild populations, and understanding the genetic underpinnings of adaptation to local environments. For individual species, expanding population genetic approaches to the whole genome will reveal large-scale structure and illuminate critical details of population interconnectedness (9). At the landscape and ecosystem levels, the availability of complete genomes for many species in an ecosystem will make approaches for investigating species presence, species interaction, or functional capacity using environmentally sourced DNA (eDNA) from water (87), sediments (88), or the atmosphere (89). These approaches will rely on the ability to map eDNA reads to large databases of well-annotated sequences. Metagenomic approaches to exploring biotic diversity diversity, such as the Tara Oceans initiative (90, 91), will be transformed by EBP genome data, again *via* large-scale read mapping to rich, open datasets. The EBP should promote significant pilot projects that explore the use of genomic resources in assessing the diversity, functional capacity, and temporal dynamics of selected ecosystems in “genomic observatories”, preferably in the Global South, through the FIF.

#### Beyond just the genome

The Encyclopedia of DNA Elements (ENCODE) project has shown the power of deep, coordinated multimodal genomic assays in discovering the function and regulation of genomes in humans and model species (92–94). Similar deep-dive functional genomics analyses of species selected for their phylogenetic disparity or for their potential to illuminate particular evolutionary transitions would be very powerful. We can imagine additional species being selected for a Diversity ENCODE program, developing new approaches to permit multimodal data generation from diverse systems.

Similarly, cell atlasing projects focused on humans and other model species have illuminated the cellular diversity of tissues and systems, revealing the genetic underpinning of complex traits such as immunity and development (95, 96). The Biodiversity Cell Atlas (BCA) initiative proposes expanding the list of species assayed at the single-cell level to explore the dynamics of development and environmental response across eukaryotes (97). The BCA will coordinate with the EBP to sample across diversity to illuminate the evolution and diversification of cell types across life and, in turn, BCA data will enrich genome annotation in targeted species and their relatives, for example, by enhancing understanding of co-expression networks and of the links between the diversity of genes present in a genome and species phenotypes.

### (5) Integrating innovative, planet-friendly informatics

Information technology (IT) strategies impact the whole span of EBP activities from organism identification to dissemination of sequence information through data portals and publications. Implementation of IT solutions on a global scale is challenged by restrictions on data access and sharing under international treaties, (such as the Nagoya Protocol), lack of standardization of metadata, different IT infrastructure capabilities and data across EBP affiliates, and different standards for data analysis, schemas, archiving, and sharing. Computation is energy-intensive, especially when AI is used (98), and it would be self-defeating for the EBP to contribute significantly to climate change-inducing emissions because of the project’s hunger for computer power. To address these challenges, EBP affiliates will need to actively promote the use of trusted global commons for laboratory protocols, computational pipelines, raw data, specimen and assembly metadata, genome sequences, and post-genomic data products. Solutions to these challenges will require that the EBP deals openly with issues of data provenance and meets the objectives of both access *and* benefits sharing.

#### Data are nothing without linked, trusted metadata

The EBP must promote consistent frameworks for collecting and accessing metadata, the information needed to track provenance, attribution, data processing activity, and public distribution, and to integrate EBP activity with global biodiversity and sequence information data services. The EBP has already taken concrete steps in planning for meeting Phase II requirements (see https://www.earthbiogenome.org/it-and-informatics-standards). Close coordination with INSDC members will be necessary to manage the archiving and sharing of public raw and analyzed data, and the EBP will promote the use of INSDC databases for all outputs. Metadata for public release must be readily combinable and interoperable, based on defined ontologies accepted in the field, and accessible through application programming interfaces. For example, information about the genetic code likely to be used by a species is needed to establish parameters for genome annotation, and sample descriptors including Darwin Core-compatible collection location, time, voucher identifiers, collector, and provenance are needed when submitting the sequence data to public repositories and when using the reference genome in the context of time- and space-resolved population datasets.

In the fast-moving EBP Phase II (over 3,000 genomes released per month, with 30,000 species “in flight” at any one time), real-time, trusted data sharing and integration will be critical. In a global project with overlapping ownership, jurisdictions, and interests, the EBP will strive to ensure the highest standards of explicit sharing of ongoing data generation. For example, GenomeArk (https://www.genomeark.org/) has been built to provide pre-publication access to and sharing of high-quality reference genomes. GoaT (https://goat.genomehubs.org) offers an integrative view of the EBP and affiliated project activities. Data partnerships among research institutions, governments, funding agencies, and the private sector will be needed to ensure the EBP delivers to its full potential. We envision long-term thinking, coordinated action, and committed funding to ensure that data sources created by the EBP will be a lasting legacy.

#### Keeping the planet green

The complexity of integrative analysis across thousands of genomes generally scales with the square of the number of species analyzed. The cost of computation (98–100) and consideration of the implied carbon footprint of the EBP favor approaches that generate shared analytic products for wide reuse. The EBP will work toward a “compute once, reuse many” approach, where core analytic products are precomputed for all to reuse. For example, whole-genome, reference-free alignment (101) is costly, with final products best shared rather than regenerated. Similarly, phylogenetic analyses of species and genes requires significant computation, and dynamically updated phylogeny and gene orthology assignments can be generated once and reused many times (102). Refactoring algorithms to support incremental updates when new species’ genomes are released—rather than re-running full analyses—can avoid costly whole-dataset recomputations. For example, in phylogenetics, heuristic placement of new taxa updates trees without recalculating from scratch (103).

Workflow management systems are critical to ensure the highest quality of data products, improve automation and scaling, reduce costs and carbon footprint, and meet FAIR and CARE principles. We envisage shared development of open resources widely distributed through workflow hubs. It is clear that AI methods will become widespread in the coming decade and, for the EBP, immediate applications in data tracking and annotation are evident. However, AI is expensive (104). We will need to ensure that EBP data are maximally AI-ready on deposition by providing detailed metadata and extensive quality-controlled training sets.

EBP projects will need to pay close attention to cybersecurity best practices in software, workflows, data storage, and management to protect data integrity and data privacy (e.g., under the Nagoya Protocol). Ensuring equitable access to all EBP data effectively requires access to global or regional cloud-based storage. Utilizing these resources effectively means that the EBP will have to maximize the compression of raw and analyzed data while ensuring carbon-neutral operation of the chosen data storage providers. Redesigning the EBP informatics workflow to minimize carbon emissions, and partnering with vendors and facilities that demonstrably reduce and offset CO_2_ from storage and computing, will lower the project’s projected carbon footprint (105).

### Building and sustaining a global EBP Phase II community

#### Democratizing genomics skills: a global approach to building a skilled workforce

EBP Phase II will require a globally distributed, well-trained, multidisciplinary workforce to address potential challenges. The skill sets required are diverse and include species identification, sample finding, collection and processing, nucleic acid extraction, and genome sequencing, assembly, curation, annotation, and analysis. In addition, the EBP needs to support the development of ethics, data governance, cultural competency, community engagement, benefit-sharing, and leadership.

The EBP will support training and mentorship activities globally, whether through sponsorship of online, open masterclasses and workshops (such as the Biodiversity Genomics Academy; https://thebgacademy.org/) or by cross-project/cross-center internship collaborations. EBP affiliates will need to build capacity by developing a skilled workforce in their geographical areas and establishing mutual aid-based training and mentoring across projects. A functioning regional node producing 1,500 reference-quality genomes per year will require at least six genomics lab technicians and four bioinformaticians, with additional support from collectors, taxonomists, and staff at natural history collections. EBP-affiliated nodes can promote capacity building within the necessary disciplines by supporting biodiversity genomics-focused components in school and university curricula. EBP affiliates should establish mechanisms to integrate Indigenous knowledge, thereby completing the virtuous circle of data sharing for capacity building.

Leadership training and mentorship will be essential, as all nodes will need to coordinate local efforts across the workflow, including community engagement, ethical and legal compliance, sample collection and processing, and generation and release of genomes. Leadership will need to coordinate globally to ensure consistency across the EBP in quality and other metrics, coordinate sampling to minimize overlap in the species being worked on, and ensure effective access and benefit-sharing. EBP representation and input at relevant global gatherings and institutions, such as the Convention on Biological Diversity (CBD) and the United Nations (UN) Climate Change Conferences, should reflect the diversity of EBP projects. The existing EBP governance structure provides initial guidance on many of these issues (https://www.earthbiogenome.org/governance-documents).

Only by establishing this global, diverse, and interconnected EBP workforce will we be able to deliver the 150,000 genomes aimed for in Phase II and build momentum to seed Phase III. Working within an EBP regional node will enable individuals to deliver impactful science and establish future careers in related areas, such as population genomics, comparative genomics, genome function and evolution, phylogenomics, conservation, human genetics, and disease. EBP Phase II can thus be an engine that delivers genomes and builds a workforce skilled in advanced bioeconomy, biotechnology, and medicine.

#### Enhancing global coordination

Organizationally, the EBP is a global network of networks. Achieving the goals of Phase II will require open, detailed coordination based on mutual respect, creative compromise, and informed agreement, meeting social, cultural, technical, scientific, and user-value goals. The EBP was established in 2018 under a Memorandum of Understanding and transitioned in 2022 to a permanent governance structure (https://www.earthbiogenome.org/governance-documents). The EBP is composed of affiliated projects that are represented on the Membership Council, a voting body that approves all EBP initiatives and actions. An elected Chair and Executive Council are charged with overall project coordination and facilitating the project’s growth. The Executive Council relies on the activities and recommendations of six standing committees (International Scientific Committee; Ethical, Legal, and Social Issues Committee; Justice, Equity, Diversity, and Inclusion Committee; Communications and Public Affairs Committee; Nominations Committee; and Governance Committee) to create policies, guidelines, and white papers, which are discussed, revised, and agreed on by the Membership Council. The International Science Committee delivers to a wide technical remit through five subcommittees: Sample Collection and Processing, Sequencing and Assembly, Genome Annotation, Data Analysis, and Information Technology. An EBP Secretariat was recently established at Arizona State University, United States, to support the completion of Phase I and the initiation of Phase II. The Secretariat coordinates business and meetings, enhances communication between members, ensures integration with affiliated projects, and reaches out to the wider public.

The need for coordination within the EBP network, which already includes 60 affiliated projects with thousands of active participants (https://www.earthbiogenome.org/affiliated-project-networks), can only grow. While all work to the same overarching objectives and standards, different affiliated projects may have distinct goals, driven by their scientific, funding, and cultural environments. EBP coordination roles have been delivered by the voluntary commitment of participants and, more recently, through multi-institutional funding for Secretariat positions. For Phase II scaling, an enhanced Secretariat is essential to link projects at both organizational and technical levels, facilitate cross-training and other synergies, support the establishment of new projects and regional nodes, integrate EBP efforts with other global biodiversity infrastructures and programs, such as GBIF, and give a voice to the EBP within international and regional policy setting fora (such as the CBD and the Agreement on Marine Biological Diversity of Areas beyond National Jurisdiction under the UN Convention on the Law of the Sea; https://www.un.org/bbnjagreement/en). This enhancement will require further acquisition of dedicated, stable funding.

Rich technical coordination between projects will be the nexus for a shared understanding and collective vision of our effort. The EBP uses GoaT to coordinate the aspirations and progress of each affiliated project (see https://goat.genomehubs.org/projects/ebp). Through GoaT, the EBP deploys effective, real-time systems to resolve species overlap between projects, support the planning of sampling campaigns, underpin the creation of distinctive funding applications, and enhance multilateral collaborations. The EBP should ensure open access to the knowledge being built, from sampling protocols to analysis methods and process management, across affiliated networks. Training programs that are open to qualified applicants everywhere are critical to developing and building global capacity in biodiversity genomics. As an example, the open Biodiversity Genomics Academy offers a self-service menu of courses and modules, dynamically updated by domain experts to reflect best practices and capture the critical details of real-world applications that can be tailored to local needs.

#### Costing the new Phase II Strategy

In 2018 we estimated that completion of the EBP would cost US$4.7 billion (1). Based on our experience and developments in laboratory technologies and informatics, we now estimate that Phase I of the EBP (sequencing and annotating the genomes of approximately 10,000 species) can be completed for US$285 million, compared to the US$600 million estimated in 2018 (Table 1). We have also re-estimated projected costs for the subsequent phases of the EBP. Based on achieving additional, reasonable efficiencies of scale and process improvements, we estimate that Phase II can deliver 150,000 high-quality genomes at one-eighth of the current unit cost of genomes in Phase I, even though we now propose sequencing all species to reference rather than short-read draft quality. Excitingly, for Phase III we estimate that all species can be sequenced to EBP reference-quality with a relatively minor (10%) increase in overall cost. With realistic assumptions about future sequencing costs continuing to decrease per species, we now estimate that genome assemblies for the vast majority of the 1.67 million named species can be completed to a uniformly high standard for US$3.9 billion. We note that these costs do not consider the variation associated with genome sequencing in the Global South and other developing areas of the world, where instrumentation and reagent costs are usually higher, but labor and sample collection costs may be lower (106).

We also propose that the EBP should commit to establishing a US$0.5 billion FIF to support research, especially in the Global South, to improve technologies for biodiversity genomics and deploy the wealth of the reference genome sequences into conservation, biodiversity enhancement, and biotechnological and biopharmaceutical applications. Thus, the full cost of the integrated EBP vision is estimated at US$4.42 billion, spread over a ten-year timeline (Table 1), with US$1.11 billion required for Phase II and US$3.1 billion for Phase III.

#### Building a global EBP funding strategy

Obtaining the US$3.9 billion required to collect, sequence, and annotate 1.67 million eukaryotic species is a considerable challenge. Phase I—sequencing 10,000 species—is decentralized to individual affiliated projects, and the larger projects have raised upwards of US$200 million. Securing funding for the completion of Phase I and initiating Phase II is a high priority and has almost been realized. Successes to date have leveraged the vision of the global project to generate considerable enthusiasm from public and private funding sources, and the EBP is pursuing multiple strategies to achieve funding goals. Ideally, the EBP requires pooled funding from multiple geographic regions to deliver improved coordination and outreach, thereby maximizing scientific and societal benefits. We recognize that funding may come with important stipulations, such as open and free access to data, considerations relating to intellectual property, benefit-sharing, capacity development and building, and partnership with Indigenous peoples and local communities, that may complicate achieving the project’s goals.

Attractive possibilities for funding include pre-competitive consortia as well as pooling resources from public agencies, major research universities and institutions, and private companies. Non-governmental organizations, not-for-profits, and the general public have shown interest in funding EBP activities. Crowdfunding among scientists has been effective in raising funds (e.g., for the VGP) and could be expanded to fill important taxonomic gaps. We anticipate that individual philanthropy will play an important role in achieving the project’s end goals, and much effort is already underway to work with visionary philanthropists who appreciate the planet-critical nature of the EBP (e.g., the Minderoo Foundation’s “OceanOmics” initiative; https://www.minderoo.org/oceanomics).

As noted above, the EBP’s impact can be maximized through rich rewards delivered by other modalities of analysis, such as deep functional genomics, proteomics, metabolomics, or single-cell atlasing, applied to a wide diversity of species. The biobanks established or enhanced for the EBP could provide essential support for these additional programs of work, which would need significant additional funding if attempted on a large scale.

Including the FIF, the total of US$4.42 billion required to fulfill the EBP goal of sequencing and analyzing 1.67 million species in 10 years is very reasonable for a global effort with such a lasting impact. The EBP offers extraordinary value for money if one compares the project cost to the US$3 billion Human Genome Project (nearly US$6 billion in inflation-adjusted dollars; see 107), the US$10 billion cost of the Webb Telescope (108), or the US$13 billion cost of discovering the Higgs boson at the Large Hadron Collider (109).

#### Justice, equality, diversity, and inclusion in access and benefit-sharing across the EBP

In delivering its mission, the EBP will need to creatively solve a series of issues arising from its commitments to justice, equality, diversity, and inclusion. These issues range from unequal infrastructure and prohibitive costs to ineffective or one-sided communication spanning all the technical and logistical challenges discussed (Table 2). The EBP acknowledges concerns surrounding access to the unprecedented volume of digital sequence information (DSI) that the project will generate, as well as the benefits that can be derived from EBP data. The EBP also recognizes the rights of countries, Indigenous peoples, and local communities that contribute to the collection of genetic resources (5), works to ensure that these rights and interests are respected and advanced throughout, and strives to cultivate a culture of working together to harness the power of DSI for the betterment of humanity. It has been estimated that Indigenous peoples and local communities steward 80% of the Earth’s remaining biodiversity (110) and thus proactive engagement with Indigenous peoples and local communities is important because the wealth of intergenerational, place-based knowledge can provide an enhanced understanding of the Earth’s biodiversity and how to protect, use, and conserve it. Recognizing the rights and interests of all these communities is crucial for the EBP to achieve its Phase II goals and lay an inclusive and equitable foundation for Phase III.

The EBP remains fully committed to sustainable development and the fair and equitable sharing of benefits arising from the use of genetic resources, operationalized through the 2014 CBD Nagoya Protocol. At the 15th Conference of the Parties (COP15) in 2022, a multilateral mechanism for benefit-sharing was included in the Kunming-Montreal Global Biodiversity Framework (111). This mechanism highlights not only monetary benefit-sharing but also the reinforcement of value creation and sharing, emphasizing inclusive and open access to DSI and the need to develop and build capacity, including technology transfer to bridge the gap between developed and developing countries. Additional international agreements, such as the UN Declaration on the Rights of Indigenous Peoples (112), as well as national laws also shape the work and ethics of the EBP.

Anticipating and addressing the ethical, legal, and social justice issues that the EBP will face during Phase II will accelerate the realization of the project’s mission. The EBP will directly contribute to the goals of the Kunming-Montreal Global Biodiversity Framework and other treaties, including the UN Convention on the Law of the Sea (113), the International Treaty on Plant Genetic Resources for Food and Agriculture (114), and the emerging Pandemic Prevention, Preparedness, and Response Agreement (115). The EBP will serve as an essential partner in enabling all nations and peoples to progress and share the benefits of global biodiversity genomics fairly and equitably. As the EBP advances into its next phases, it aims to establish mechanisms that ensure that the use of DSI leads to tangible benefits for countries, communities, and peoples. These mechanisms will include exploring models for non-monetary benefit-sharing, such as capacity development and building initiatives, technology transfer, and the development of partnerships that promote sustainable development in regions of origin, the retention of young researchers, and ensuring that the FIF is well subscribed to and equitably disbursed.

The EBP will contribute to several UN Sustainable Development Goals (SDGs) that relate to biodiversity and genetic diversity. Specifically, reference genomes will contribute to SDG 2 (Zero Hunger), SDG 14 (Life Below Water), and SDG 15 (Life on Land). EBP genomes can be directly used as primary indicators of genetic diversity (e.g., runs of homozygosity) or as a basis for cost-efficient technologies (e.g., eDNA monitoring) to monitor genetic diversity in populations over time (supporting the achievement of SDGs 14.4.1, 15.5.1, and 15.9.1). The latter will be extremely useful for conservation programs and management of marine and terrestrial resources, including animal and plant breeding (SDGs 2.5.1 and 2.5.2).

### The way forward

Based on our experience in Phase I of the EBP, in Phase II we propose sequencing 150,000 species—representing at least half of all known genera—to reference quality over 4 years. To build momentum for the remaining 90% of named eukaryotic species, we propose that Phase II includes collecting an additional 10% of the planet’s fungi, protists, plants, and animals, biobanking them for sequencing in the early years of Phase III and safeguarding them for future research.

As the EBP proceeds, it will strive to be inclusive and equitable, recognizing the biodiversity richness of the Global South by establishing and sustaining genomics capacity, particularly in countries with high biodiversity. It is vitally important to build a genomic commons where data are accessible and can be shared frictionlessly. It is also critical to show and enhance the utility of the data by driving high-impact demonstration projects in conservation, biodiversity assessment, biopharmaceutical discovery, and bioproduct identification. By sequencing phylogenetic breadth as a driver, we will span the full eukaryotic tree of life, and by diving deep into specific taxonomic groups, or complete local ecosystems, we will demonstrate the riches that could come with Phase III sequencing of all eukaryotic biota.

The revised estimated cost of Phase II of the EBP is US$1.1 billion, including US$0.25 billion from the new FIF; this is down from the US$1.6 billion estimate made in 2018. Securing funding is one of the most pressing tasks faced by the EBP. By attracting funds that facilitate the sustainable establishment of biodiversity genomics within institutions in developing economies, we will be able to not only robustly deliver Phase II but also generate proof of concept for Phase III, “sequencing all life for the future of life”. Understanding the origins and evolution of life on Earth is a human pursuit equivalent to understanding the origins and evolution of the universe. Beyond this, the wealth of practical applications that will emerge from sequencing eukaryotic life, ranging from conservation to climate adaptation and ecosystem preservation, likely makes the EBP the most ambitious and beneficial project in the history of science.

## Supplementary Material

Supplementary Material

## Figures and Tables

**Table 1:** Estimated budget for the Earth BioGenome Project.

	Phase I	Phase II	Phase III	Total
	Most families	Most genera	All species	
**No. of species to be sampled and sequenced**				
Sampled	10,000	300,000	1,360,000	**1,670,000**
Sequenced	10,000	150,000	1,510,000	**1,670,000**
**Project costings (US$, millions)**				
Sample collection (collection, identification, shipping, and biobanking)	10	250	690	**950**
Sequencing (genomic and transcriptomic), assembly, annotation, and databasing	250	500	2,000	**2,750**
Research and development for collection, sequencing, and informatics*		100	100	**200**
Coordination and secretariat funding	5	6	10	**21**
**Project core cost (US$, millions)**	**265**	**856**	**2,800**	**3,921**
Foundational Impact Fund (FIF)		250	250	**500**
**Total with FIF (US$, millions)**	**265**	**1,106**	**3,050**	**4,421**
Original proposal (US$, millions)	637	1,612	2,493	4,742
Cost per reference genome (US$)	26,500	6,100	1,900	2,400**

* We assume that the technology providers will continue to increase capacity and quality and decrease the per-sample cost of genomic data acquisition, as they have over the last three decades. We do not include these research and development costs here.

** Overall cost per reference genome based on estimated costs.

**Table 2:** Technical and social challenges the Earth BioGenome Project (EBP) must face.

Technical and social challenges	Issues	Progress required
Sample collection and processing	Unequal distribution of biobanking/vouchering infrastructure Diverse permissions regulations leading to inequitable species collection and inadequate metadata	Connect providers to existing infrastructure for sample deposition Create and enforce policies ensuring fair attribution Generate sustainable sample shipment mechanisms Enforce transparency and compliance, and reduce duplicated efforts
Sequencing	Unequal infrastructure distribution and costs Differing technological equipment and reagent storage costs Prohibitive infrastructure upkeep costs for sustained participation in the Global South	Lobby for local infrastructure Seek discounted service rates Prepare hands-on sequencing training
Assembly and annotation	Unequal access to resources Limited accessibility and/or restricted access due to paywalls Deprioritization of curation in laboratories in the Global South	Design all code, workflows, and standards as open access Deploy software utilizable in resource-limited settings (e.g., CPU/GPU use) Deliver hands-on assembly, curation, and annotation training using open-access content Develop tools to handle data in compressed formats
Downstream analysis	Unequal infrastructure distribution and costs Biased datasets for training models Computationally intensive Unequal capacity to translate genomics into applications	Develop and use downstream analysis tools responsibly and sustainably Reduce species biases and sequencing duplication Promote international collaboration
Workforce and training	Funding for capacity building and knowledge transfer Unidirectional knowledge sharing Lack of diversity in STEM	Scale equitable and inclusive training models Support career paths for underrepresented groups Provide reciprocal bidirectional training with local partners Invest in project coordination and communication
Engagement	Unfair distribution of benefits/burdens Distrust from legacy extraction and exploitation Inadequate engagement with trans-sectoral interested parties and inclusion of their worldviews throughout the project	Prioritize cultural awareness and inclusion of other worldviews and value systems Engage internationally with communities throughout the research process Strengthen existing partnerships and co-build new ones Obtain appropriate consent and mutually agreed-upon terms before project onset
Communication and coordination	Disparities in digital technologies and high-speed internet access Priorization of, and balance between, FAIR and CARE principles Ineffectiveness of global communicational coordination	Engage with policymakers in Global South nations Reconcile FAIR and CARE principles Invest in centralized outreach and coordination Dismantle power imbalances where possible

Note: The suggested solutions are non-exhaustive and many apply to multiple stages of each work category.

Abbreviations: **CARE,** collective benefit, authority to control, responsible, ethical; **CPU/GPU,** central processing unit/graphics processing unit; **FAIR,** findable, accessible, interoperable, and reusable; **STEM,** science, technology, engineering, and medicine.

## Data Availability

The original contributions presented in the study are included in the article/Supplementary Material. Further inquiries can be directed to the corresponding authors.

## BOX 1 Genomes from a box.

Responses to health emergencies such as the West African Ebola virus epidemic and the global COVID-19 pandemic have demonstrated that mobile facilities—built off-site, shipped in transport containers, and ready for use once connected to local electricity and water supplies—can transform crisis response. Our response to the biodiversity crisis needs a similar approach. One of the major hurdles to establishing genomics in a new location is the sourcing of the tools and skills required to run a genomics laboratory. One “leapfrog” solution might be if prospective Earth BioGenome Project (EBP) regional nodes, collaborative funders, and established genome centers worked together to source funding and build and deliver a genomics laboratory, with equipment ready-to-install and with training, reagent supply, and support guaranteed. This model is already in operation for biodiversity work, for example, in the In Situ Laboratory Initiative (https://insitulabs.org/).

A genomics laboratory in a box, or gBox, could be one route to establishing the approximately 25 EBP regional nodes needed to deliver Phase II. We envisage the gBox arriving as a standard shipping container, equipped to act as a high-throughput genomics laboratory. Inside would be all the equipment needed to transform specimens into DNA and RNA, make sequencing libraries, produce long- and short-read sequence data at scale, and turn those data into assembled and annotated genomes.

However, a gBox would be more than just hardware. It would also contain the equipment needed to collect, identify, and store species, ready to be placed in a field laboratory. Laptop computers and digital cameras would be ready to record specimens and their metadata, which would be stored in local databases before being transmitted to global systems, such as Global Biodiversity Information Facility and International Nucleotide Sequence Database Collaboration. An initial tranche of reagents would ship with the gBox, with subsequent shipments available on demand. The computer system would come ready-loaded with best-in-class pipelines developed elsewhere in the EBP and with a commitment to keep these up to date. The collaborating established centers would also offer training in sampling and recording, extraction and other molecular biology operations, instrument operation and use, and transforming raw data into chromosome-scale reference genomes. Training and support would be ongoing, creating a shared virtual laboratory to enable real-time problem solving. All sites with a gBox could participate in a virtual genomics commons to co-learn skills and pass on discoveries.

We intend to build a consortium of reagent suppliers and equipment manufacturers that would support the global collection of gBox labs with guaranteed reagent costing and onsite technical support. This partnership would ensure that the gBoxes run to their full potential. We expect each team, comprising six local laboratory staff members and a similar number of bioinformaticians to be able to deliver at least 1,500 genomes (of ~1 Gbase each) per year.

The gBox would be owned and operated by the receiving institute, which would be free to use it for other sequencing needs, such as urgent viral sequencing for public health or post-genome projects funded by the EBP Foundational Impacts Fund. The EBP will search for visionary funders who seek to partner with us to sponsor the manufacture, shipping, installation, and four-year operational costs of a fleet of gBoxes at EBP regional nodes.
